# Consistency of Eating Rate, Oral Processing Behaviours and Energy Intake across Meals

**DOI:** 10.3390/nu9080891

**Published:** 2017-08-17

**Authors:** Keri McCrickerd, Ciaran G. Forde

**Affiliations:** 1 Clinical Nutrition Research Centre (CNRC), Singapore Institute for Clinical Sciences (SICS), Agency for Science, Technology and Research (A*STAR), National University Health System, Singapore 117599, Singapore; Keri_McCrickerd@sics.a-star.edu.sg; 2 Department of Physiology, Yong Loo Lin School of Medicine, National University of Singapore, Singapore 117593, Singapore

**Keywords:** eating rate, meal size, oral processing, consistency

## Abstract

Faster eating has been identified as a risk factor for obesity and the current study tested whether eating rate is consistent within an individual and linked to energy intake across multiple meals. Measures of *ad libitum* intake, eating rate, and oral processing at the same or similar test meal were recorded on four non-consecutive days for 146 participants (117 male, 29 female) recruited across four separate studies. All the meals were video recorded, and oral processing behaviours were derived through behavioural coding. Eating behaviours showed good to excellent consistency across the meals (intra-class correlation coefficients > 0.76, *p* < 0.001) and participants who ate faster took larger bites (*β* ≥ 0.39, *p* < 0.001) and consistently consumed more energy, independent of meal palatability, sex, body composition and reported appetite (*β* ≥ 0.17, *p* ≤ 0.025). Importantly, eating faster at one meal predicted faster eating and increased energy intake at subsequent meals (*β* > 0.20, *p* < 0.05). Faster eating is relatively consistent within individuals and is predictive of faster eating and increased energy intake at subsequent similar meals consumed in a laboratory context, independent of individual differences in body composition.

## 1. Introduction

Fast eating has been identified as a risk factor for increased energy intake [1], obesity, and metabolic disease [2,3], and researchers have recommended eating slower to protect against excess food intake, by chewing more, taking smaller or fewer bites, and/or pausing in between mouthfuls [4,5,6,7,8]. However, while observational studies link self-reported fast eating to higher BMI and obesity risk [2,9,10,11,12], laboratory measures of actual eating behaviours suggest the relationship between oral processing and weight status is less clear. Some studies have reported that individuals with overweight or obesity tend to eat faster by taking more frequent and larger bites and/or chewing less than lean individuals [5,13,14,15], while others have observed few or inconsistent differences in eating behaviours [5,16,17,18,19]. Most recently, Fogel et al. showed that faster eating in children was underpinned by larger bite size and less chewing per gram of food, independent of the child’s weight status [20], though children who ate faster did consume more energy at a single meal and were more likely to be classed as overweight.

If faster eating is a risk factor for overweight, one prediction is that faster eating should be relatively consistent within an individual and linked to increased energy intake across multiple meals. In support of this, several early studies have characterised individuals based on the extent to which eating rate decelerated towards the end of a meal and reported good test–retest reliability for measures of within-meal eating microstructure, using both video recordings of bite and chewing behaviours [21,22] and Universal Eating Monitor (UEM) technology [8,23,24]. Though based on small participant numbers, this earlier work indicates that people who eat faster at one meal eat faster at the same meal consumed on another day. Yet studies reporting that faster eating rate promotes increased energy intake only compared behaviours at a single eating occasion [1]. Since energy intake at a meal is also influenced by a series of other related factors (e.g., reported appetite [25], food texture, and palatability [26]), it is unclear whether faster eaters are likely to consistently eat more across multiple meals, and whether eating rate at one meal is likely to be a good predictor of intake at another.

The current research draws on data collected across four studies conducted recently in the Clinical Nutrition Research Centre (CNRC) to assess individual consistency in eating rate, oral processing behaviours, and food intake across multiple laboratory-based meals. In all of the studies, participants attended four identical test days to consume four similar meals ad libitum and have their eating behaviours recorded. Specifically, the research aimed to:

1. Determine the consistency of eating behaviours (defined as eating rate, oral processing, and energy intake) within individuals.
2. Describe the relationship between eating rate, oral processing behaviours, and energy intake across multiple meals.
3. Test whether eating rate at one meal can be used to predict eating rate and energy intake at subsequent meals.

Understanding whether eating behaviours measured at single eating occasion are likely to reflect eating behaviours at subsequent meals, within the context of the laboratory, has implications for a better understanding of the proposed relationship between oral processing behaviours, energy intake, and weight status.

## 2. Materials and Methods

### 2.1. Participants

Participants were 146 male and female volunteers taking part in one of four studies running at the Clinical Nutrition Research Centre (CNRC) between July 2015 and September 2016. Study-specific methods are reported in detail elsewhere [27,28,29]. Each study followed similar recruitment criteria and procedure. Eligible participants were healthy males (Studies 1–4) and females (Study 4 only) between 21–50 years, without allergies or aversions to the study foods, and not using any medication that could influence appetite or energy metabolism. Participant characteristics are presented in Table 1. There was some variation in participants’ age, percentage fat mass, and reported restrained eating across the four studies, though these were all within a typical range. All research activities were granted ethical approval by the Singapore National University Hospital Domain Specific Review Board, and the data were collected in accordance with the Declaration of Helsinki.

### 2.2. Test Meals

**Figure 1:**
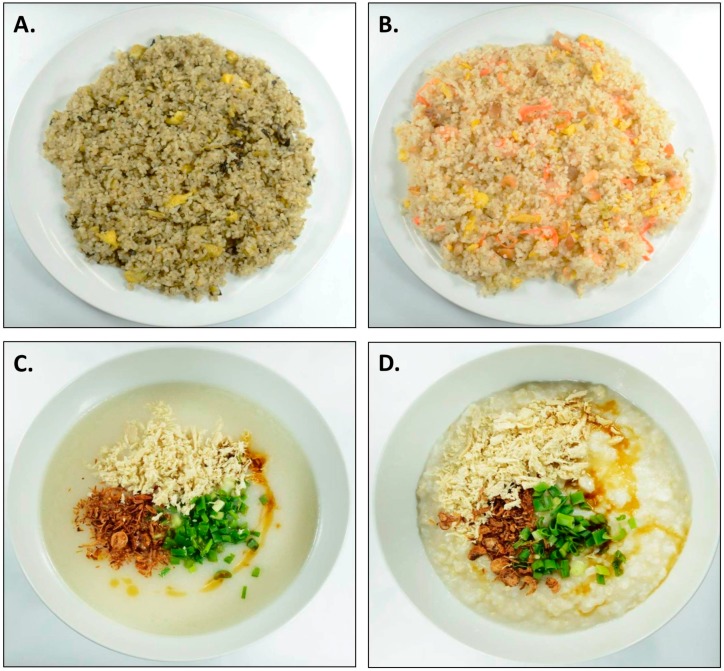
The four test meals consumed in each study. (**A**) Participants in Study 1 consumed olive fried rice; (**B**) Participants in Studies 2 and 3 consumed Yang Chow fried rice; Participants in Study 4 consumed (**C**) thin and (**D**) thick chicken rice porridge varying in energy density.

The rice-based test meals presented in Figure 1 differed across the studies. These were comprised of familiar and acceptable foods commonly consumed in Singapore and served alongside a glass of water (250 mL). In Studies 1–3, the participants were served the same test meal across the four days. In Study 1, this consisted of 1000 g vegetarian fried rice (1.89 kcal/g, Figure 1A). In Studies 2 and 3, participants were served 800 g yang chow fried rice (1.57 kcal/g, Figure 1B) across the four days. Both were supplied by a local catering company (JR Foods, Singapore) and reheated from frozen immediately before serving according to the manufactures instructions. In Study 4, the test meals were rice-based porridges which varied in texture (thick/chewy vs. thin/less chewy, Figure 1C,D, respectively) and energy density (lower vs. higher), producing four variations that were served across the test days: thin/0.57 kcal, thin/1.01 kcal/g, thick/0.57 kcal/g, and thick/1.01 kcal/g. The porridge recipes are described in detail elsewhere [27]. The porridge base consisted of a combination of brown and white rice, chicken stock, maltodextrin, and sunflower oil, with the following standardised toppings: shredded chicken, scallions, fried shallots, sesame oil, and soy sauce. All ingredients were commercially available. The thin texture manipulation was achieved by grinding the rice to powder and adding more chicken stock during cooking, and were consumed at a significantly faster rate than the thicker versions across participants (82.5 vs. 141.2 g/min [27]). The differences in energy density were achieved by varying the quantities of maltodextrin and oil, but participants were unaware, and energy density did not influence eating rate [27]. The porridges were prepared fresh on the test day and served in 1000 g portions with toppings added immediately before serving.

### 2.3. Oral Processing Behaviours

Eating rate and oral processing of the test meals were determined by coding video recordings of participants during consumption, using previously established methods and described fully in recent papers [27,31]. A coding scheme was developed for use alongside the behavioural annotation software ELAN (version 4.9.1, Max Planck Institute for Psycholinguistics, The Netherlands: [32]) to code the frequency of every bite, chew, and swallow taken within each of the four meals, starting with the first bite and ending with the final swallow. From this, the time the food spent in the mouth (total oral exposure time in seconds) and the time spent with no food in the mouth (inter-bite interval in seconds) was derived. These oral processing behaviours were then used in combination with the weight of the food consumed to define the total eating rate (g/min), average bite size (g/bite), oral exposure time per bite (s), and number of chews taken per gram of food (chews/g).

All behavioural coding was completed by trained researchers. Validation coding was conducted for a minimum of 10% of the total coded videos and had to show at least 80% agreement between all coders for the data to be accepted for analysis.

### 2.4. General Procedures

Data were collected across four studies investigating the effect of food/beverage structure and energy density on satiation and satiety responses (rated appetite and subsequent intake). Participants believed they were taking part in studies investigating the effects of “food on mood”, and no participants took part in more than one of the studies.

All participants completed a questionnaire assessing their health and dietary habits. Basic anthropometry was collected during screening (Studies 1–3) or upon study completion (Study 4). Each study required participants to consume four test meals ad libitum, either for lunch (Studies 1–3) or breakfast (Study 4) on four non-consecutive days with at least three days between each session. All participants fasted from 11 pm the evening before a study day.

All ad libitum test meals were served in individual air-conditioned booths at the CNRC and followed the same procedure across all studies. Each booth was fitted with a computer (Hewlett Packard Notebook PC 11- d020TU, Singapore) and a webcam (Logitech HD c310, Singapore) mounted above eye level on the wall facing the subject. Participants were informed of the video recording during the consent process, but were unaware that the recording was used to assess oral processing behaviours. Participants were unable to see themselves on the video display, and the video was started as they were seated. Participants began the session by completing pre-meal appetite ratings disguised as “Mood Questions”. To do this, ratings of *hunger*, *fullness*, and *desire to eat* were presented on 100-point visual analogue scales (VAS) alongside distractor “mood” ratings of *happy*, *stressed*, *alert*, *energetic*, and *clearheaded*. The VAS question was “How <rating> do you feel right now”, anchored from *Not at all* <*rating*> (0) to *Extremely* <*rating*> (100). All ratings were presented in a randomised order and completed on the computer.

The test meals were served immediately after completion of the first set of appetite ratings, and participants were prompted to taste the food and rate its pleasantness using the same VAS format. Participants were then instructed “now you may eat as much or as little of the <test meal> as you like to feel comfortably full.” Participants were allocated 20 min to eat, and those who finished the portion within this time were provided with another immediately. The test meal was removed as soon as the participant indicated to the researcher that they were finished, at which point participants completed a set of post-meal appetite ratings. The total weight of food consumed was calculated by measuring the weight of the serving plate/bowl (i) empty before serving; (ii) full after serving; and (iii) at the end of the meal. All measurements were taken on a Sartorius balance accurate to 0.001 g.

### 2.5. Analysis

Intra-class correlation coefficients (ICCs) with 95% confidence intervals (CIs) were used to determine the consistency of Eating Rate (g/min), Oral Processing Behaviours (average bite size, oral exposure per bite, number of chews per gram), and Energy Intake (kcal) within individuals across the four test meals (i.e., if participants at a faster rate in Meal 1, do they also eat faster at Meals 2–4?). Two-way random error were assumed, and an ICC value of <0.5 indicates poor consistency, 0.50–0.75 indicates moderate consistency, 0.75–0.9 indicates good consistency, and >0.90 excellent consistency [33].

Multiple regression analyses were used to test the relationship between eating rate, energy intake and oral processing characteristics across the four meals. Preliminary analyses revealed a main effect of the Study on the relationship between eating rate and energy intake, due to the differences in texture and energy density in the study foods, so for clarity these data are reported separately for each of the four studies. These analyses controlled for meal pleasantness (a significant independent predictor of energy intake) and included “Eating Behaviour × Meal” interaction terms, which tested whether the relationships were consistent across different test days within the studies. Additional analyses were conducted to further control for other individual differences linked to eating rate and/or energy intake across the meals within this sample: BMI, % Fat Mass, Age, pre-meal appetite (specifically rated fullness, as this was the only appetite measure significantly linked to intake and eating rate), sex (for Study 4 only) and reported restrained eating. For all analyses, standardised (*β*) and unstandardized (*b*) betas are presented with bootstrapped 95% CIs and *p*-values.

Finally, similar linear models, controlling for meal pleasantness, were used to assess whether measured eating rate at one meal (Meal 1) could be used to predict eating behaviours at the other meals (Meals 2–4). All analyses were conducted in IBM SPSS Statistics version 23.

## 3. Results

### 3.1. Consistency of Eating Rate, Oral Processing, and Energy Intake within Individuals

The ICCs are reported in Table 2 and indicated good to excellent consistency in eating rate (g/min), oral processing behaviours, and energy intake across the four meals and studies (all significant at *F* ≥ 7.42, *p* < 0.001). This means, for example, that individuals who ate at a faster rate had a larger bite size or consumed more did so consistently across the four meals within each study.

### 3.2. Relationship between Eating Rate, Oral Processing, and Intake over Multiple Meals

**Figure 2:**
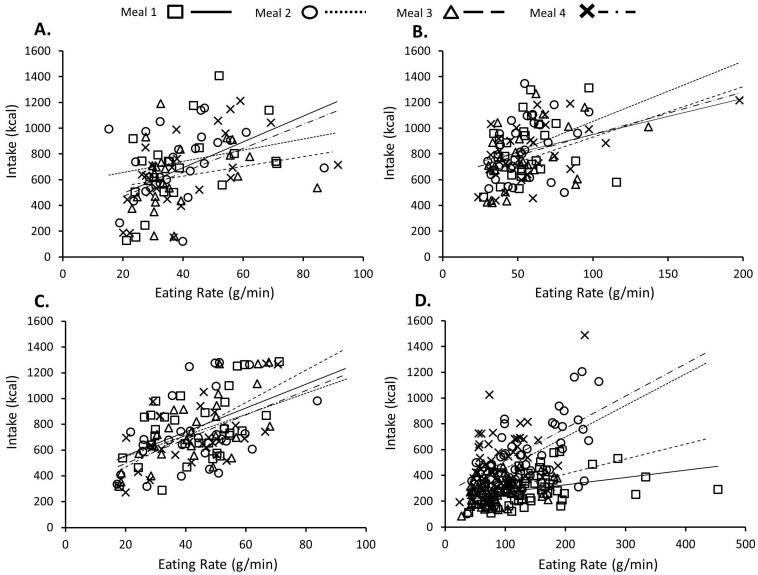
Scatter plots of individual eating eates and energy intake across each of the four test meals consumed in (**A**) Study 1; (**B**) Study 2; (**C**) Study 3; and (**D**) Study 4. The lines represent the regression line of best fit for each meal.

Figure 2A–D shows the relationship between eating rate (g/min) and energy intake (kcal) at each meal for each of the four studies. Controlling for meal day and rated pleasantness, a faster eating rate significantly predicted larger intake in Study 1 (*β* = 0.28, *b* = 5.26 [1.80, 10.36], *p* = 0.022), Study 2 (*β* = 0.31, *b* = 2.85 [1.32, 4.47], *p* = 0.002), Study 3 (*β* = 0.54, *b* = 9.83 [6.79, 12.60], *p* = 0.001) and Study 4 (*β* = 0.33, *b* = 1.18 [0.66, 1.89], *p* = 0.001). The strength of the relationship between eating rate and intake was consistent across all four meals in Studies 1 to 3 (*p* ≥ 0.340 for all Eating Rate ×Meal interaction terms), but not in Study 4 when the meals varied in texture and energy density (see Figure 2D). Specifically, the relationship between eating rate and intake on Day 1, when the thin lower energy porridge was consumed, was significantly weaker than the relationship seen in Meal 2 (*p* < 0.001) and Meal 4 (*p* = 0.001), where the two higher energy porridges (thin and thicker, respectively) were consumed, but was marginally similar to the relationship seen in Meal 3, when the thicker lower energy porridge was consumed (*p* = 0.056). After controlling for individual characteristics (BMI, % Fat Mass, Age, pre-meal fullness, sex, and reported restrained eating), the relationships between eating rate and intake remained significant across all of the studies and meals (*β* ≥ 0.17, *b* ≥ 0.62, *p* ≤ 0.025).

Across all studies and meals, faster eating was characterised by participants taking larger bites (*β* ≥ 0.39, *b* ≥ 1.72, *p* < 0.001). Having a shorter oral exposure time per bite was independently associated with faster eating in Studies 2–4 (*β* ≤ −0.72, *b* ≤ −1.58, *p* ≤ 0.002), and marginally in Study 1 (*β* = −0.37, *b* = −0.60 [−1.44, −0.21], *p* = 0.079). Fewer chews per gram of food consumed predicted faster eating in Study 1 (*β =* −0.58, *b* = −12.28 [−16.09, −6.78], *p* = 0.001) and 3 (*β* = −0.29, *b* = −4.47 [−6.46, −2.59], *p* < 0.001), but not Study 2 (*β* = 0.04, *b* = 1.63 [−10.19, 9.29], *p* = 0.787) and 4 (*β* = −0.08, *b =* −9.62 [−25.06, 5.62], *p* = 0.240). Increased energy intake was also predicted by larger bite size across all studies (*β* ≥ 0.24, *b* ≥ 11.36, *p* ≤ 0.018) and shorter oral exposure per bite in Studies 3 and 4 (*β* ≤ −0.38, *b* ≤ −14.96, *p* ≤ 0.033), but not Studies 1 and 2 (*β* = −0.14, *b* ≤ −5.02, *p* ≥ 0.099). Fewer chews per gram did not predict energy intake across any of the studies (*β* ≥ −0.18, *b* ≥ −65.62, *p* ≥ 0.197). All relationships were consistent across meals and remained after controlling for additional individual characteristics (BMI, % Fat Mass, Age, pre-meal fullness, sex, and reported restrained eating).

### 3.3. Eating Rate at One Meal as a Predictor of Eating Behaviours at Subsequent Meals

**Figure 3:**
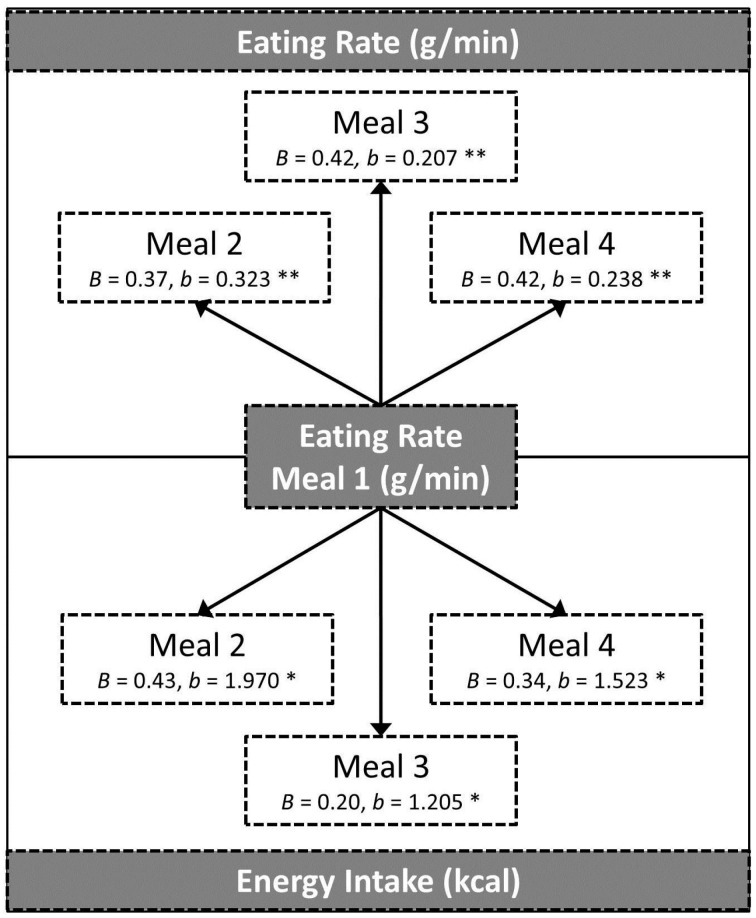
Standardised (*B*) and unstandardized (*b*) regression coefficients for the relationship between eating rate at Meal 1 and Eating rate and Energy intake at all the other meals, controlling for meal pleasantness and study. * *p* < 0.05, ** *p* < 0.001.

Figure 3 summarises the influence of eating rate (g/min) at Meal 1 as a predictor of eating rate and intake at subsequent meals (Meals 2–4). Controlling for meal pleasantness and Study, faster eating at Meal 1 significantly predicted faster eating and greater energy intake in all of the subsequent meals within each study.

## 4. Discussion

Consistent with the research aims, we showed that faster eating rate was (i) consistent within individuals from meal to meal; (ii) repeatedly associated with increased energy intake; and (iii) predicted faster eating and increased energy intake at other meals. This was independent of the study, test day, and individual differences in meal pleasantness, appetite, body composition, and reported eating style.

A number of studies have now linked a faster rate of eating to increased energy intake within a single meal [1], and measures of eating rate and cumulative energy intake using UEMs [8,23,24] and behavioural coding [21,22] have shown good test–retest reliability of measured oral processing behaviours across multiple meals—particularly for initial eating rate within a meal [24]. Our findings extend these earlier studies to show that eating behaviours coded from webcam recordings were also consistent within individuals and linked to subsequent energy intake; participants who ate faster and consumed more energy at one meal tended to do so at the others, and faster eating was consistently associated with larger bite size and shorter oral processing time per bite. Importantly, eating rate at the first meal significantly predicted eating rate and energy intake not just at that meal, but at the other three meals, suggesting that one-time coded eating behaviours could be a relatively accurate representation of behaviours at other laboratory meals, where the same or similar food is consumed.

Though rate of eating appears to be a consistent eating behaviour within individuals, food texture is also an important determinant of oral processing, impacting eating rate and bolus formation, independent of the person consuming the food. For instance, harder, thicker, and chewier foods require more chewing, spend more time in the mouth, and are consumed at a slower rate than softer, less chewy foods [31,34]. The influence of food texture on individual differences in eating rate and intake could not be assessed in Studies 1–3, as participants repeatedly consumed the same meal. However, in Study 4, the rice meals varied in both texture (thicker/chewier vs. thin/less chewy) and energy density (higher energy density vs. lower energy density). In that study, the thicker porridges required more chewing and were consumed at a slower rate and in smaller quantities compared to the thin versions, independent of how much the porridge was liked, whereas energy density had no impact on eating rate, leading participants to consistently consume the most from the higher energy density versions [27]. The current reanalysis of these data shows that faster eating was associated with increased intake across all of the meals (see Figure 2D), and this relationship was strongest when the higher energy density porridges were consumed and was less affected by differences in the texture of the food. Together these findings suggest that a fast eater who consistently consumed foods that are high in energy density and fast to eat could be particularly susceptible to overconsuming at meal times. More generally, faster eaters took larger bites and were quicker than slower eaters at reducing these into a swallowable bolus, possibly by chewing more per gram of food consumed and/or having a lower threshold for acceptable bolus properties, like food particle size. It is possible that the effects of faster eating on increased energy intake might be curtailed by consuming less energy dense foods as well as those that are harder and chewier, which promote oral processing and a slower eating rate.

Understanding how individual differences in eating rate interact with features of the foods a person consumes to influence “real-world” energy intake is key to unpacking the link between the reported faster eating and higher BMI and obesity risk noted previously in observational studies [2,9,10,11,12]. However, it is clear that individual differences in laboratory-measured eating behaviours like bite size and eating rate also occur independently of overweight and obese weight status. Participants in the current studies were predominantly non-dieting and non-obese young males with BMIs and percent fat mass in a typical range, yet faster eating was still independently associated with larger bite size and increased energy intake across all of the meals. This supports new evidence that faster eating in children was underpinned by larger bite size and less chewing per gram of food, independent of weight status [20]. One limitation in the current data was the predominance of male participants, so we could not directly test for sex differences in the consistency of eating behaviours in a balanced way. However, we have previously identified no differences between male and female participants in the extent to which eating rate impacted intake across four meals [27]. Another limitation is that eating behaviours were only measured in the laboratory and with a limited range of foods, and more research is required to confirm whether eating rate measured in this way predicts eating behaviours outside of the laboratory. It is possible that an “obesogenic eating style” of consistently faster eating and taking larger bites can exist across a range of individuals, but may only be associated with overweight when combined with a number of other dietary characteristics known to increase energy intake at a meal, such as higher energy density foods [35,36,37], softer food textures [27,38], and large portion size [39]. A better understanding of the link between faster eating and increased energy intake in the context of these wider dietary behaviours will improve behavioural targets for weight management.

## Figures and Tables

**Table 1:** Participant characteristics (Mean ± SD and Range) across the studies.

*N* = 146	Study 1	Study 2	Study 3	Study 4		*p* ^3^
	Male (*n* = 26)	Male (*n* = 30)	Male (*n* = 34)	Male (*n* = 27)	Female (*n* = 29)	
**Age (years)**	27 ± 5 ^a^ (21–37)	28 ± 5 ^a^ (21–41)	29 ± 7 ^a^ (21–48)	25 ± 3 ^a,b^ (21–36)	23 ± 3 ^b^ (21–39)	0.001
**BMI (kg/m^2^)**	23 ± 3 (18–29)	22 ± 2 ^a^ (19–25)	22 ± 2 ^a^ (18–25)	23 ± 3 ^a^ (18–28)	21 ± 3 ^a^ (16–29)	0.043
**Fat Mass (%) ^1^**	21 ± 7 ^b^ (7–34)	18 ± 8 ^b,c^ (3–33)	16 ± 4 ^c^ (8–24)	17 ± 5 ^b,c^ (9–27)	29 ± 6 ^a^ (18–43)	0.001
**Dietary Restraint (0–100%) ^2^**	36 ± 21 ^a,b^ (0–81)	33 ± 25 ^b^ (0–81)	35 ± 23 ^a,b^ (0–95)	52 ± 21 ^a^ (11–100)	46 ± 27 ^a,b^ (6–95)	0.012

^1^ Measured using Tanita Bio-Impedance Analyser BC-418. ^2^ Measured using the revised Three Factor Eating Questionnaire [30], where 0% represents low dietary restraint and 100% represents the highest score of restraint. ^3^ Between-groups ANOVA for the main effect of participant group on individual characteristics. Within each row, values with different letters: a, b, and c are significantly different (*p* < 0.05 for Bonferroni-corrected comparisons), while values with the same letter are not significantly different (*p* > 0.05). BMI = body mass index.

**Table 2:** Intra-class correlation coefficients (ICC) with 95% confidence intervals for eating behaviours across the four sessions within each study.

Eating Behaviour	Study 1	Study 2	Study 3	Study 4	Combined
**Intake (kcal)**	0.97 [0.94, 0.98]	0.94 [0.90, 0.97]	0.96 [0.92, 0.98]	0.88 [0.82, 0.92]	0.95 [0.94, 0.96]
**Eating rate (g/min)**	0.95 [0.90, 0.98]	0.87 [0.75, 0.94]	0.95 [0.91, 0.98]	0.77 [0.65, 0.86]	0.87 [0.83, 0.91]
**Bite size (g)**	0.81 [0.62, 0.92]	0.84 [0.70, 0.92]	0.95 [0.91, 0.98]	0.76 [0.63, 0.85]	0.80 [0.74, 0.85]
**Oral exposure per bite (s)**	0.82 [0.64, 0.92]	0.97 [0.94, 0.99]	0.94 [0.88, 0.97]	0.83 [0.74, 0.89]	0.93 [0.74, 0.89]
**Chews per gram (chews/g)**	0.95 [0.96, 0.98]	0.96 [0.92, 0.98]	0.96 [0.93, 0.98]	0.85 [0.77, 0.90]	0.96 [0.95, 0.97]

An ICC of <0.50 indicates poor consistency, 0.50–0.75 indicates moderate consistency, 0.75–0.90 indicates good consistency, and >0.90 excellent consistency [33].
